# Hypertension artérielle pulmonaire au cours de la sclérodermie: à propos de 12 cas

**Published:** 2012-01-18

**Authors:** Maboury Diao, Mouhamadou Bamba Ndiaye, Adama Kane, Malick Bodian, Nadége Christelle Tchintchui, Alassane Mbaye, Mouhamadoul Mounir Dia, Moustapha Sarr, Assane Kane, Serigne Abdou Ba

**Affiliations:** 1 Hôpital Aristide Le Dantec, avenue Pasteur, Dakar, Sénégal; 2 Hôpital Général de Grand Yoff, Dakar, Sénégal

**Keywords:** Hypertension artérielle pulmonaire, sclérodermie, Sénégal

## Abstract

**Introduction:**

La survenue de l’hypertension artérielle pulmonaire (HTAP) est un tournant dans l’évolution de la sclérodermie. L’objectif de cette étude est de décrire les aspects épidémiologiques et évolutifs de l’HTAP au cours de la sclérodermie systémique.

**Méthodes:**

Nous avons réalisé une étude descriptive concernant des patients suivis pour sclérodermie systémique, au service de Dermatologie de l’hôpital Aristide Le Dantec entre Janvier 2000 et Août 2009. Ces patients étaient inclus dans l’étude après exploration cardio-vasculaire (ECG, échocardiographie-Doppler). Nous avons étudié les paramètres épidémiologiques, cliniques, paracliniques et évolutifs des patients.

**Résultats:**

Nous avons enregistré 12 cas d’hypertension artérielle pulmonaire parmi les 83 patients atteints de sclérodermie systémique soit une prévalence de 14,45%. L’âge moyen des patients était de 43,58 ans±12,5 ans et le sex-ratio (H/F) de 0,33. Sur le plan clinique, la dyspnée était quasi constante (75%) et la douleur thoracique présente dans 25% des cas. Le syndrome de Raynaud était observé chez 8 patients soit 66,67% de nos patients. L’électrocardiogramme montrait des signes de surcharge droite chez 4 malades (33,33%) et la radiographie thoracique en faveur d’une fibrose pulmonaire chez 4 patients. L’échocardiographie-Doppler notait une insuffisance tricuspide importante dans 58, 33% des cas (7 patients), une pression artérielle pulmonaire systolique (PAPs) en moyenne de 66,25±29,3 mmHg, une dilatation des cavités cardiaques droites dans 5 cas et un mouvement paradoxal du septum interventriculaire chez 3 malades (33,33%). Il était également noté 3 cas (25%) d’épanchement péricardique. Nous avons déploré 4 décès (33,33%).

**Conclusion:**

L’hypertension artérielle pulmonaire est une complication fréquente et grave de la sclérodermie. Son dépistage, grâce à l’échocardiographie-Doppler systématique, constitue une étape fondamentale de la prise en charge.

## Introduction

La sclérodermie systémique est une maladie du tissu conjonctif interstitiel et vasculaire associée à des anomalies du système immunitaire, conduisant à une fibrose extensive et une oblitération vasculaire [1]. L’hypertension artérielle pulmonaire (HTAP) constitue l’une des principales causes de mortalité des patients atteints de sclérodermie systémique particulièrement de ses formes limitées. Elle survient le plus souvent tardivement dans l’évolution de la maladie et connait plusieurs mécanismes [2].

En Occident, sa fréquence est estimée à environ 16% [3], par contre en Afrique, les études sont rares avec des fréquences disparates. Notre étude a pour objectif d’étudier les aspects épidémiologiques et évolutifs de l’HTAP au cours de la sclérodermie systémique.

## Méthodes

Nous avons réalisé une étude transversale descriptive concernant une cohorte de patients hospitalisés ou suivis en ambulatoire pour sclérodermie systémique, au service de Dermatologie de l’hôpital Aristide Le Dantec de Dakar entre le 01 Janvier 2000 et le 31 Août 2009. Ces patients avaient bénéficié d’une exploration cardio-vasculaire au service de Cardiologie du même hôpital. Les patients hospitalisés ou suivis à titre externe, atteints de sclérodermie systémique dont le diagnostic était confirmé selon les critères de l’American College of Rheumatology (ACR) [4] et qui avaient bénéficié d’une échocardiographie Doppler, étaient inclus.

Nous n’avions pas inclu les patients atteints de sclérodermie systémique n’ayant pas bénéficiés d’une échocardiographie Doppler, les patients atteints de sclérodermie localisée, les patients chez qui le diagnostic de sclérodermie systémique n’était pas confirmé et les situations d’HTAP sans sclérodermie systémique.

Les paramètres étudiés étaient : les données épidémiologiques, les antécédents (obstétricaux, circonstances de découverte de la maladie, notion d’exposition aux solvants organiques, existence de cas familiaux de sclérodermie systémique et d’HTAP), l’existence ou non de diabète, d’HTA, d’asthme, d’une affection cardiaque préexistante (valvulopathie ou une autre atteinte), de prise d’anorexigènes. Nous avons en outre apprécié les données cliniques (dont les signes fonctionnels cardio-vasculaires, dermatologiques), les signes généraux (constantes hémodynamiques, état général), les signes d’examen physique des appareils et systèmes.

Les données paracliniques biologiques (créatininémie, protéinurie des 24 heures, bilan immunologique), électrocardiographiques, radiographiques, échocardiographiques, fonctionnelles respiratoires étaient étudiées de même que le traitement.

L’évolution était appréciée sur la durée d’hospitalisation, la ré-hospitalisation, le suivi à court, moyen et long terme et sur la survenue de complications fatales ou non. Sur le plan statistique nous avons élaboré une fiche d’enquête qui a servi de support aux données. La saisie et l’analyse des données ont été effectuées sur le logiciel SPSS 17.0 (Statiscal Package For Social Sciences) for windows.

## Résultats

**Figure 1:**
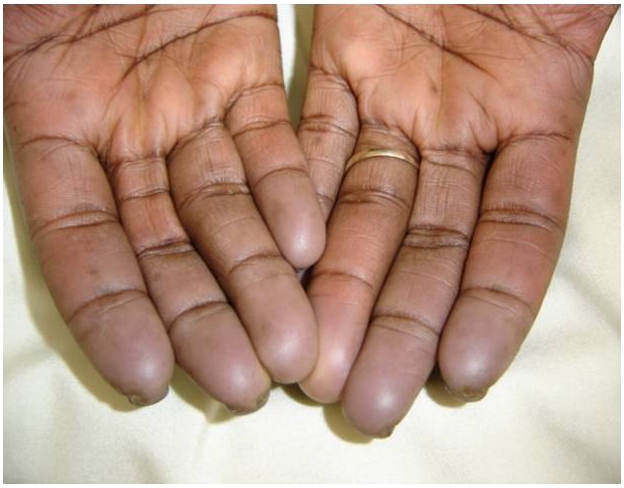
Phénomène de Raynaud à la phase asphyxique

Durant la période d’étude, nous avions colligé 142 cas de sclérodermie dont 83 avaient bénéficié d’une échocardiographie Doppler parmi lesquels 12 présentaient une HTAP, soit une prévalence hospitalière de 14,45%. L’âge moyen était de 43,58±12,5 ans (extrêmes de 22 et 60 ans) avec une prédominance féminine (9 femmes pour 3 hommes). La durée moyenne d’hospitalisation était de 27,8± 14 jours (extrêmes de 11 et 64 jours), la durée moyenne d’évolution de 4,6±2,1 années (extrêmes de 4 mois et de 20 ans). Les antécédents étaient représentés par l’hypertension artérielle retrouvée chez deux patients. Il n’y avait pas de cas de prise d’anorexigènes ni d’antécédent familial de sclérodermie et d’HTAP. La symptomatologie fonctionnelle était dominée par la dyspnée stade II à IV (9 cas) et le syndrome de Raynaud (8 cas). Les signes digestifs étaient représentés par une dysphagie et un reflux gastro-oesophagien respectivement dans 3 cas. L’examen dermatologique avait mis en évidence: des macules hypochromiques en « moucheture » chez 9 malades, une sclérose cutanée chez 8 malades (66,66%), une alopécie chez 5 patientes (41,67%), des ulcérations et des cicatrices stellaires pulpaires des doigts de la main et des pieds compliquant le syndrome de Raynaud (Figure 1) chez 4 malades (33,33%). L’examen cardio-vasculaire notait une arythmie auscultatoire chez 4 patients (33,33%), un éclat de B2 pulmonaire chez 7 patients (58,33), un souffle systolique xyphoïdien dans 5 cas (41,67) et des signes d’insuffisance cardiaque droite dans 5 cas (41,67). A la biologie on notait une créatininémie élevée dans deux cas et la protéinurie de 24 heures était positive chez 3 patients.

Les anticorps antinucléaires retrouvés sont résumés dans le Tableau 1. Les anticorps anticentromères avaient été retrouvés dans 16,67% des cas, en association avec les anticorps anti B2GP1 dans 1 cas (il s’agit de l’anticorps du syndrome des antiphospholipides). Les anticorps anti Scl70 étaient notés chez 1 patient. Le bilan immunologique n’était pas déterminé chez 7 patients.

**Tableau 1:** Répartition des patients en fonction des anticorps antinucléaires

Anticorps	Nombre de patients	Pourcentage
Anticentromères	1	8,33
Anticentromère+anti B2GP1	1	8,33
Scl70	1	8,33
Absence d’Ac anti-ECT	2	16,67
Indéterminé	7	58,33

Les anomalies électrocardiographiques étaient notées chez 8 malades (66,66%). Il s’agissait de troubles de rythme chez 5 malades (41,67%), d’hypertrophie auriculaire droite dans 3 cas et ventriculaire droite dans 4 cas. La radiographie du thorax avait mis en évidence une cardiomégalie chez 6 patients (50%), une dilatation de l’artère pulmonaire dans 5 cas, des images de fibrose pulmonaire dans 4 cas. La fuite tricuspidienne, constamment retrouvée à l’échocardiographie Doppler, était importante dans 58,3% des cas (Tableau 2).

**Tableau 2:** La répartition des malades en fonction de la pression artérielle pulmonaire systolique

Pression artérielle pulmonaire systolique	Malades	Pourcentage
30-45 mmHg (légère)	4	33,33
46-59 mmHg (modérée)	3	25
≥ 60 mmHg (sévère)	5	41,67

**Figure 2:**
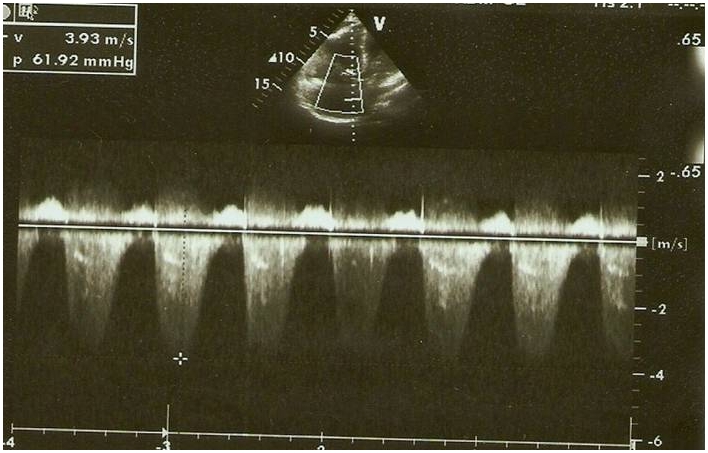
Image d’échocardiographie mode 2D, incidence 4 cavités associée au Doppler continu mettant en évidence une HTAP sévère (PAPs à 66 mmHg)

**Figure 3:**
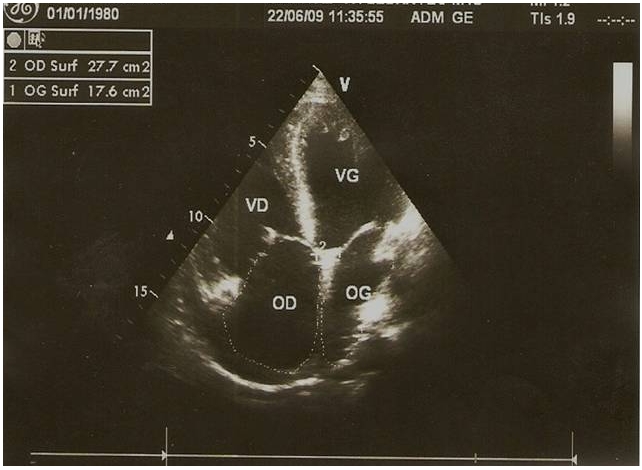
Image d’échocardiographie mode 2D, incidence 4 cavités mettant en évidence une dilatation de l’oreillette droite

**Figure 4:**
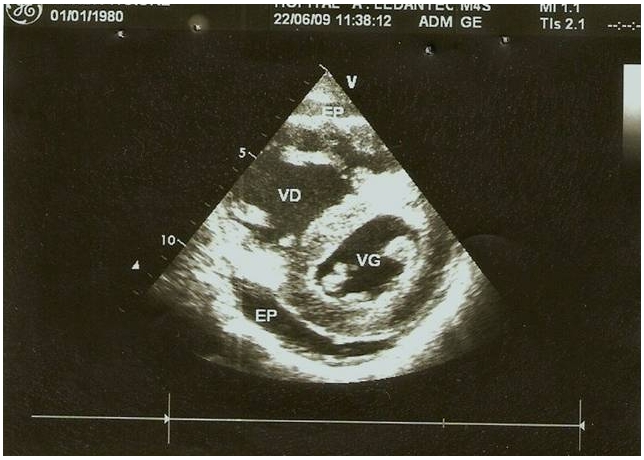
Image d’échocardiographie mode 2D, petit axe, transventriculaire montrant un épanchement péricardique circonférentiel de moyenne abondance

**Figure 5:**
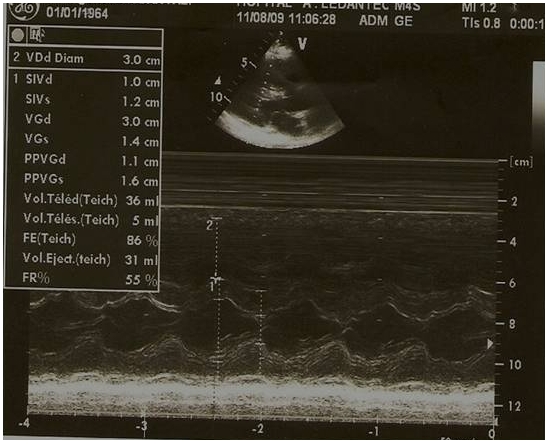
Image d’échocardiographie mode TM, coupe parasternale grand axe, incidence transventriculaire montrant une dilatation modérée du ventricule droit

La pression artérielle pulmonaire systolique (PAPs) moyenne était de 66,25±29,3 mmHg (extrêmes de 35 et de 132 mmHg) Figure 2. L’évaluation de la fonction ventriculaire droite par le TAPSE (tricuspid annular plane systolic excursion) avait trouvé un TABSE moyen de 17,2 mm (extrêmes de 12 mm et de 22 mm), un TAPSE normale (supérieur ou égale à 15 mm) dans 3 cas, bas (inférieur à 15 mm) dans 2 cas et indéterminée chez 7 patients. La dilatation des cavités cardiaques droites (Figure 3, Figure 4, Figure 5) était notée dans 41,67% des cas et l’épanchement péricardique chez 3 patients (Figure 4). L’exploration fonctionnelle respiratoire (EFR) retrouvait un syndrome obstructif dans deux cas, un syndrome restrictif et un syndrome mixte respectivement chez un patient.

Les pathologies associées étaient une dermatomyosite, une thyroïdite d’Hashimoto et un syndrome des antiphospholipides. Sur le plan évolutif on notait quatre décès (33,3%) en rapport avec une mort subite (1 cas), une insuffisance cardiaque droite terminale (2 cas) et une insuffisance rénale chronique terminale associé à une cardiomyopathie dilatée (1 cas). Le suivi des malades avait permis d’enregistrer 4 cas de réhospitalisation. Le suivi était régulier chez 5 malades et 4 malades étaient perdus de vue.

## Discussion

La prévalence hospitalière de l’HTAP au cours de la sclérodermie systémique dans notre série est de 14,45%. Cette prévalence apparaît variable d’une étude à l’autre. Elle dépend en effet des méthodes de dépistage employées ainsi que des cohortes étudiées. Elle est de 5 % dans le travail de Battle [5], de 13% (prévalence cumulée) dans l’étude de MacGregor [6] portant sur une cohorte de 152 patients, toutes ces deux études étant basées sur un dépistage échocardiographique. Les données épidémiologiques françaises montrent que la prévalence minimale de I′HTAP associée à la ScS est de l′ordre de 7,8% [7]. En Afrique, les données sont encore pauvres à cause de la rareté des descriptions portant sur l’HTAP. Nous avons constaté un âge de survenue jeune dans notre travail comme l’a signalé Humbert dans son étude [8] avec un âge moyen de 48±17 ans. Cependant en Occident, elle apparait beaucoup plus tôt [9,10]. De même la prédominance féminine est en général retrouvée dans la littérature [5,9–11]. Ceci s’expliquerait par le fait que le terrain de prédilection de la sclérodermie systémique est le sexe féminin [6,8,12]. Par contre la durée moyenne d’évolution de la sclérodermie systémique au moment de la découverte de l’HTAP est variable de 4,6±2,1 ans dans notre étude, MacGregor et al [6] retrouvaient 6,4 ans et pour d’autres auteurs elle était supérieure à 10 ans [10,12,13]. Au plan clinique la dyspnée d’effort est le symptôme le plus fréquent de l’HTAP [10], suivi du syndrome de Raynaud (66,67% de nos patients) alors que pour la plus part des auteurs, il représente l’un des premiers signes de la maladie [9,14]. Les macules hypochromiques en moucheture sont également fréquentes [9]. Sur le plan immunologique le taux significatif des anticorps anti-centromères et des anticorps anti-Scl70 diffère selon les auteurs [6,12]. Cependant il n y a pas de corrélation entre la présence d’anticorps et la survenue d’HTAP [10,15,16]. L’échocardiographie Doppler était l’examen de référence pour le dépistage de l’HTAP dont la sévérité est variable en moyenne de 54 mmHg [9,10,12]. Les autres anomalies échocardiographiques dans l’HTAP sont représentées par la dilatation des cavités droites, un mouvement paradoxal du septum interventriculaire et une altération de la fonction systolique du VD [17,9]. En ce qui concerne l’épanchement péricardique, les études échographiques rapportent une prévalence variant entre 16% et 41% [17–19].

La précocité du diagnostic est un paramètre clé du pronostic puisque celui-ci est directement corrélé au degré d’élévation de la pression artérielle pulmonaire [6]. Ainsi, en raison de la fréquence et de la sévérité de l’HTAP associée à une ScS, certaines sociétés savantes préconisent la pratique annuelle d’une échocardiographie-Doppler en cas de ScS, même en l’absence de symptôme évocateur d’HTAP [20,21]. Il s’agit de l’examen de choix pour le dépistage de l’HTAP dans la ScS, mais le cathétérisme cardiaque droit reste l’examen de référence pour la confirmation diagnostique [22]. La réduction de la capacité de transfert du CO associée à des volumes pulmonaires normaux est reconnue dans la littérature comme un signe précoce et évocateur de l’hypertension artérielle pulmonaire [10,13,15,23]. Owens [23] a ainsi montré qu’une diminution isolée de la DLCO n’existe que rarement (5%) chez les patients sans hypertension artérielle pulmonaire. La survenue d’une HTAP au cours de la ScS constitue un tournant évolutif de la maladie grevant ainsi cette affection d’une lourde mortalité.

Sur le plan de la mortalité, nos résultats se rapprochent de ceux de Hachulla [24] qui avait observé 32% de décès chez les patients porteurs d’une HTAP.

## Conclusion

L’HTAP constitue l’une des complications les plus graves de la sclérodermie systémique. Elle survient le plus souvent tardivement dans l’évolution de la maladie, avec une nette prédominance féminine. L’échocardiographie Doppler est l’examen de choix pour dépister l’HTAP. Sa réalisation est indiquée devant toute suspicion clinique; elle doit être systématique, une fois par an.
